# *ARID1A* Mutations in Gastric Cancer: A Review with Focus on Clinicopathological Features, Molecular Background and Diagnostic Interpretation

**DOI:** 10.3390/cancers16112062

**Published:** 2024-05-30

**Authors:** Giuseppe Angelico, Giulio Attanasio, Lorenzo Colarossi, Cristina Colarossi, Matteo Montalbano, Eleonora Aiello, Federica Di Vendra, Marzia Mare, Nicolas Orsi, Lorenzo Memeo

**Affiliations:** 1 Department of Medicine and Surgery, Kore University of Enna, 94100 Enna, Italy; giuangel86@hotmail.it; 2 Department of Medical, Surgical Sciences and Advanced Technologies G.F. Ingrassia, Anatomic Pathology, University of Catania, 95123 Catania, Italy; giulioatta@gmail.com; 3 Pathology Unit, Department of Experimental Oncology, Mediterranean Institute of Oncology, 95029 Catania, Italy; lorenzo.colarossi@grupposamed.com (L.C.); cristina.colarossi@grupposamed.com (C.C.); eleonora.aiello@grupposamed.com (E.A.); 4 PhD Program in Precision Medicine, University of Palermo, 90144 Palermo, Italy; 5 Department of Chemical, Biological and Environmental Chemistry, University of Messina, 98122 Messina, Italy; 6 Medical Oncology Unit, Department of Experimental Oncology, Mediterranean Institute of Oncology, Viagrande, 95029 Catania, Italy; 7 Leeds Institute of Medical Research, St James’s University Hospital, The University of Leeds, Leeds LS9 7TF, UK; n.m.orsi@leeds.ac.uk

**Keywords:** *ARID1A*, gastric cancer, SWI/SNF complex, PD-L1, microsatellite instability, PARP inhibitors

## Abstract

**Simple Summary:**

*ARID1A* mutations are emerging as a prognostic and predictive factor in gastric cancer. Recent studies suggest their potential role in predicting patient response to novel treatment strategies including immunotherapy, poly(ADP) ribose polymerase (PARP) inhibitors, mammalian target of rapamycin (mTOR) inhibitors, and enhancer of zeste 2 polycomb repressive complex 2 subunit (EZH2) inhibitors. The aim of the present review is to provide a detailed appraisal of the significance of the loss of *ARID1A* functionality in GCs, and examine its prognostic and therapeutic implications.

**Abstract:**

AT-rich interaction domain 1 (*ARID1A*) is a pivotal gene with a significant role in gastrointestinal tumors which encodes a protein referred to as BAF250a or SMARCF1, an integral component of the SWI/SNF (SWItch/sucrose non-fermentable) chromatin remodeling complex. This complex is instrumental in regulating gene expression by modifying the structure of chromatin to affect the accessibility of DNA. Mutations in *ARID1A* have been identified in various gastrointestinal cancers, including colorectal, gastric, and pancreatic cancers. These mutations have the potential to disrupt normal SWI/SNF complex function, resulting in aberrant gene expression and potentially contributing to the initiation and progression of these malignancies. *ARID1A* mutations are relatively common in gastric cancer, particularly in specific adenocarcinoma subtypes. Moreover, such mutations are more frequently observed in specific molecular subtypes, such as microsatellite stable (MSS) cancers and those with a diffuse histological subtype. Understanding the presence and implications of *ARID1A* mutations in GC is of paramount importance for tailoring personalized treatment strategies and assessing prognosis, particularly given their potential in predicting patient response to novel treatment strategies including immunotherapy, poly(ADP) ribose polymerase (PARP) inhibitors, mammalian target of rapamycin (mTOR) inhibitors, and enhancer of zeste 2 polycomb repressive complex 2 subunit (EZH2) inhibitors.

## 1. Introduction

According to the American Cancer Society estimates, there were 26,500 new diagnoses and 11,130 deaths attributable to stomach cancer in the US in 2023 alone, accounting for around 1.5% of new diagnoses of malignancy [1]. Gastric adenocarcinoma accounts for about 95% of gastric cancer (GC) cases and exhibits high morphological and molecular heterogeneity [2,3,4]. The high mortality rate of GC is mainly explained by the fact that most cases are diagnosed as late-stage disease and the existing lack of effective treatments, which has driven research endeavors into the molecular mechanisms driving the disease [2,3,4].

The Cancer Genome Atlas (TCGA) and the Asian Cancer Research Group (ACRG) performed a whole genome analysis study to investigate genomic alterations in gastric tumors [5]. Based on genetic, epigenetic, and gene expression profiles, four distinct GC subtypes have been identified: Epstein–Barr virus (EBV) positive, microsatellite instability (MSI), genomically stable (GS), and chromosomal instability (CIN) [6]. This novel classification reflects the wide molecular heterogeneity of GCs but also holds crucial prognostic and therapeutic implications [7,8,9,10]. However, taking into account the high turn-around times and the costs of the whole genome analysis used in TCGA classification, immunohistochemistry (IHC) and EBV-RNA in situ hybridization (EBER-ISH) have emerged as alternative surrogates for molecular classification in daily clinical practice [7,8,9,10,11,12,13].

Recent studies have focused on AT-rich interaction domain 1A (*ARID1A*) as a new molecular driver gene in GC [14,15]. *ARID1A* is a component of the SWItch/sucrose non-fermentable (SWI/SNF) chromatin remodeling complex, which dynamically alters chromatin structure and orchestrates gene expression [14,15,16,17]. *ARID1A* is frequently mutated in GC, with mutation rates ranging from 14% to 24% [17,18,19]. The predominant types observed are nonsense and frameshift mutations, which result in either functional or expression abnormalities in the *ARID1A* protein [17,18,19]. Consequently, the absence of *ARID1A* protein expression may serve as an indicator of the mutation status in the *ARID1A* gene [14,15,16,17,18,19]. In this regard, numerous studies have demonstrated the link between *ARID1A* expression and several clinicopathological features of GC [14,15,16,17,18,19].

*ARID1A* plays a key role in promoting tumorigenesis principally through three mechanisms: increased proliferation, disrupted differentiation, and suppression of apoptosis [14,15,16,17,18,19]. Notably, in GCs as well as other tumors, a correlation exists between *ARID1A* and phosphatidylinositol-4,5-bisphosphate 3-kinase catalytic subunit α (PIK3CA) mutations [20,21,22,23,24,25,26,27,28]. In detail, PIK3CA mutations are linked to the EBV subtype in the TCGA classification and are associated with microsatellite-stable (MSS)/TP53 and microsatellite instability (MSI)-High subtypes in the ACRG classification [8,19,20]. These subtypes also exhibit a higher prevalence of *ARID1A* loss-of-function mutations [8,19,20]. The aim of the present review is to provide a detailed appraisal of the significance of the loss of *ARID1A* functionality in GCs and examining its prognostic and therapeutic implications.

## 2. Biological Functions of *ARID1A*

**Figure 1:**
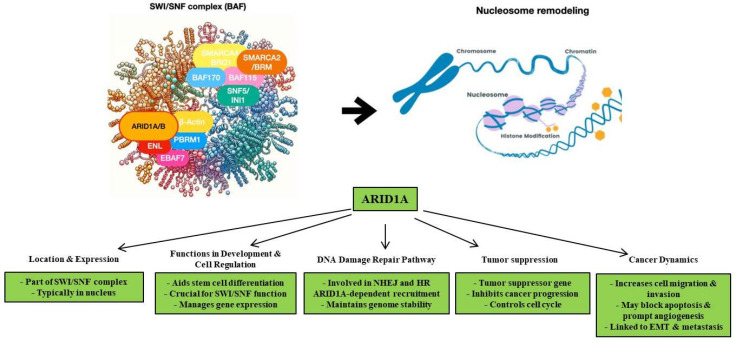
This image illustrates the role of ARID1A in the SWI/SNF complex, highlighting its role in chromatin remodeling and gene expression.

All relevant findings concerning *ARID1A* status in gastric cancer have been summarized in Figure 1 and Table 1 and Table 2.

As a SWI/SNF complex component, *ARID1A* is typically located in the nucleus and is strongly expressed across various tissue types [14]. *ARID1A* expression plays a role both in the development and regulation of cell function, thus fulfilling a range of biological activities [51,52]. More specifically, *ARID1A* plays a central role in regulating the differentiation of stem cells, including cardiac progenitor, neural stem/progenitor, and embryonic stem cells [51,52]. The absence of *ARID1A* results in the destruction/loss of function of the SWI/SNF complex, which in turn leads to an imbalance in the expression of genes involved in cell stemness and differentiation [51,52]. Recent findings have also indicated that *ARID1A* is essential in the two main DNA damage repair pathways: non-homologous end joining (NHEJ), which occurs mainly in the S phase of the cell, and homologous recombination (HR), which occurs primarily in the G1 and G2 phases [47]. The balanced development of the two repair pathways keeps the genome stable. In this context, the recruitment of the SWI/SNF complex ATPase subunit to sites of the DNA damage site depends on *ARID1A* [47]. Thus, inhibition or loss of *ARID1A* leads to the inactivation of the NHEJ pathway. Moreover, *ARID1A* interacts with the phosphatidylinositol (PI)3/PI4 kinase family proteins which are essential for HR-mediated responses [47].

*ARID1A* is generally considered to be a tumor suppressor gene that can inhibit the biological behavior of malignant tumors and regulate the cell cycle to promote apoptosis to exert its anticancer effects [14,47,53]. Several studies on cancer cell lines demonstrated that *ARID1A* inhibition promotes the migration and invasion of neoplastic cells, inhibits apoptosis, and induces angiogenesis [14,47,53,54]. Additionally, other studies have demonstrated that *ARID1A* inhibition induces epithelial–mesenchymal transition (EMT) and promotes tumor cell metastasis [55,56].

## 3. *ARID1A* Mutations in Gastric Cancer

*ARID1A* mutations have recently emerged as a key event in the pathogenesis of GC [47]. Following TP53, this gene is the second-most mutated in this setting, with mutations detected in 8–27% of cases [32]. The intriguing aspect of these mutations is their variation and distribution across different GC subtypes [32,33,34]. Notably, *ARID1A* mutations were predominantly found in the EBV-positive subtype, suggesting a specific pathway of disease development in this group [35]. Building upon this molecular landscape, in 2015, the ACRG reclassified gastric cancer into four distinct subtypes to better direct treatment and prognosis [57,58]. These subtypes are MSI, MSS/EMT, MSS/TP53+, and MSS/TP53−. The mutation rates of *ARID1A* in these subtypes were: 44.2% in MSI, 13.9% in MSS/EMT, 18.6% in MSS/TP53+, and 5.9% in MSS/TP53− [57,58]. In this scenario, recent studies have highlighted that the deletion and/or mutation of *ARID1A* increases the efficiency of EBV infection in gastric epithelial cells, linking genetic alterations in GC with viral infection, and suggesting potential novel avenues of therapeutic intervention [59,60]. Moreover, these alterations pose challenges in recruiting mismatch repair proteins, thereby initiating the development of specific subtypes like EBV-positive and MSI subtypes of gastric cancer [59,60].

Setia et al. further simplified the classification of gastric cancer using immunohistochemistry and in situ hybridization, identifying subtypes such as EBV-positive, MSI-high, and variations based on E-cadherin and P53 expression [44]. Their work also demonstrated that EBV-positive and MSI-high gastric cancers generally show a better prognosis compared to other histotypes [44].

The relationship between *ARID1A* mutational status and GC also has significant implications for immunotherapy. The loss of *ARID1A* protein expression in GC inversely correlates with the positive expression of MSI-H subtype and PD-L1. Since these two latter subtypes respond more effectively to immune checkpoint inhibitors (ICIs), *ARID1A* expression represents a potential biomarker for guiding immunotherapy in GC [45,46]. Notably, ARID1A expression appears to play a crucial role in modulating the tumor microenvironment and influencing the response to immunotherapy in gastric cancer (GC). In detail, ARID1A expression in GC appears to be closely linked with several biomarkers that influence response to immunotherapy. In this regard, the upregulation of PD-L1 expression, association with higher TMB levels, and potential as a predictive biomarker suggest that ARID1A status could overcome the limitations of classical biomarkers and provide valuable insights into patient stratification for immunotherapy [8,19,20,44].

## 4. Clinical and Prognostic Significance of *ARID1A* Mutation in Gastric Cancer

While Zhou et al. have reported lower *ARID1A* protein expression levels in GCs compared to normal gastric tissue, recent studies suggest that complete or partial loss of *ARID1A* expression is associated with both reduced progression-free survival (PFS) and overall survival (OS) in patients with GC [15,20,22,29,36,37,38,39]. In this respect, Wang et al. performed an immunohistochemical and molecular study of 272 primary GCs, where *ARID1A* protein deletion emerged as an independent risk factor of poor prognosis [29]. More specifically, a correlation between *ARID1A* deletion and critical clinico-pathological parameters including tumor differentiation, lymph node metastasis, and tumor size has also been demonstrated [36]. However, other studies challenge the notion that an absence of *ARID1A* protein expression is a marker of poor prognosis [29,30,39]. For instance, Ibarrola–Villava and co-workers have reported that patients with absent *ARID1A* expression had a significantly higher OS compared to those with positive expression profiles [30]. Similarly, in a cohort study utilizing tissue microarrays of 173 GCs, no clear relationship emerged between OS and the loss of *ARID1A* expression [31]. The reasons behind these conflicting findings could be explained by multiple factors, including intra-tumoral heterogeneity, the limited sample sizes in existing studies, and potential variations in immunohistochemical procedures, including the sensitivity of detection methods and the non-standardized interpretative criteria used.

## 5. Molecular Pathways Involved in *ARID1A* Mutation

Recent research has shed light on the interaction between *ARID1A* and TP53 in gastric cancer [40,41,42,43]. Studies have demonstrated that silencing *ARID1A* in GC cells in vitro leads to a decrease in the expression of two downstream target genes of TP53 [40,41,42,43]. This finding suggests that *ARID1A* and TP53 may influence the transcription of certain target genes, thereby inhibiting tumor growth [40,41,42,43]. This hypothesis aligns with previous research conducted by Guan in the field of ovarian cancer, further strengthening the proposed synergy between *ARID1A* and P53 in cancer suppression [41].

Although clinical studies in this area are limited, emerging evidence suggests that the loss of *ARID1A* expression could serve as a biomarker for protein kinase B (AKT) pathway activation and might predict the effectiveness of AKT inhibitors in patients with GC [40,41,42,43]. In this regard, Zhang et al. reported that knocking out *ARID1A* in GC cell lines in vitro directly impacts the transcription of 3-phosphoinositide-dependent protein kinase-1 (PDK1) and phosphatidylinositol-4,5-bisphosphate 3-kinase (PIK3CA) within the PIK3/AKT pathway [40]. This alteration leads to phosphorylation changes in key components of the PIK3/AKT signaling pathway, including AKT and the mammalian target of rapamycin (mTOR) [40]. Further supporting these findings, other in vitro studies have confirmed that in *ARID1A*-deficient GC cells, the PI3K/AKT pathway is activated, promoting the proliferation of GC cells [61]. Of note, these *ARID1A*-deficient cells showed increased sensitivity to inhibitors targeting PI3K and AKT [61].

## 6. Therapeutic Approach in *ARID1A*-Deficient Gastric Cancer

New therapeutic paradigms, such as targeted therapy, immunotherapy, and anti-angiogenic therapy have recently emerged as alternative and potentially useful approaches for the management of GC (Table 3) [47]. Immunotherapy with immune checkpoint inhibitors, especially PD-1/PD-L1 inhibitors, has generated considerable interest in recent clinical trials due to their efficacy in the treatment of solid tumors [47]. However, a significant proportion of GC patients remains unresponsive to such interventions, underscoring the urgent need to identify reliable biomarkers to identify patients who could benefit most from immune checkpoint blockers (ICBs) [47]. In this regard, the KEYNOTE-059 trial demonstrated that pembrolizumab was more effective in treating gastric or gastroesophageal junction (GEJ) adenocarcinoma with a PD-L1 combined positive score (CPS) of 1 or higher [62]. Similarly, the CHECKMATE-649 trial showed that nivolumab, in combination with chemotherapy, improved OS in advanced GC and EGJ cancers compared to chemotherapy alone, particularly in cases where PD-L1 CPS was five or higher [63]. A pivotal factor in this context is the tumor mutation burden (TMB), which gauges a tumor’s ability to generate neoantigens and predicts the effectiveness of immunotherapy across various tumor types [47]. Defects in mismatch repair (dMMR), typically arising from mutations in mismatch repair protein-encoding genes, lead to a microsatellite instability-high (MSI-H) status [47]. Patients with MSI-H/dMMR tumors show significant responses to immunotherapy, as highlighted by studies including the KEYNOTE-016, 164, 012, 028, and 158 trials [47]. As a result, pembrolizumab received US Food and Drug Administration (FDA) approval for treating metastatic or unresectable solid tumors bearing dMMR or MSI-H biomarkers [64]. In addition, the degree of tumor-infiltrating lymphocytes (TILs) has been recognized as a potential biomarker for predicting the success of PD-1/PD-L1 immunotherapy [65]. Interestingly, *ARID1A* expression in GC is closely aligned with these biomarkers which influence response to immune blockade therapy [47]. The loss of *ARID1A* in GC inversely correlates with PD-L1 expression. *ARID1A* deficiency has been shown to upregulate PD-L1 expression by activating the PI3K/AKT/mTOR pathway [66,67]. Moreover, bioinformatics studies have suggested that gastrointestinal cancers with *ARID1A* mutations exhibit higher TMB levels and thus may benefit from immunotherapy [67]. Thus, the discovery of a link between *ARID1A* deletion and the profile of immunotherapy biomarkers (PD-L1, TMB, MMR, and TILs) in GC suggests the possible role of *ARID1A* deletion as a predictive biomarker for responses to immunotherapy. Another recent discovery is the reported sensitivity of *ARID1A*-deficient tumors to poly(ADP ribose) polymerase (PARP) inhibitors [48,68]. However, the efficacy of PARP inhibitor monotherapy in cancers lacking *ARID1A* is somewhat limited, often requiring combination therapy for enhanced effectiveness [69]. For example, the combination of the PARP inhibitor olaparib with the PI3K inhibitor BKM120 has shown promising results as a potential treatment strategy for *ARID1A*-deficient GC [69]. Moreover, recent studies have identified *ARID1A* expression as a marker to identify GC patients who may benefit from mTOR inhibitor therapy [47]. Inhibition of the PI3K/AKT pathway has also been shown to enhance the sensitivity to tumor-specific CD8+ T cell-mediated cytotoxicity [69,70]. In light of these findings, combining PI3K/AKT/mTOR inhibitors with ICIs (including PD-1 and CTLA-4 inhibitors or other forms of immunotherapy) appears to offer patients an avenue for effective treatment [47,69,70]. Another promising area of research involves the enhancer of zeste 2 polycomb repressive complex 2 subunit (EZH2), an enzymatic catalytic subunit of polycomb repressive complex 2 (PRC2), which is frequently overexpressed and aberrantly regulated in several tumors [47,49,50]. Targeting EZH2 with specific inhibitors is particularly relevant in *ARID1A*-mutated cancers since EZH2 is known to influence tumor-infiltrating lymphocytes, thereby contributing to creating an immunosuppressive tumor microenvironment that facilitates immune evasion by tumor cells [47,49,50]. By inhibiting EZH2, existing immunotherapies may be enhanced, leading to more effective treatment [47,49,50]. However, given the documented risk of inflammatory and autoimmune system complications, a deeper understanding of the interplay between EZH2 inhibitors and ICB in treating *ARID1A* mutated cancers remains crucial [47,49,50]. Collectively, these findings open the way for more tailored and potentially effective treatment strategies for GC. However, understanding the intricate relationships between these biomarkers and patient stratification for suitability to receive immunotherapy efficacy requires further research to enhance the survival prospects of those receiving ICB therapy.

## 7. *ARID1A* Immunohistochemistry in Gastric Cancer

The loss of *ARID1A* expression evaluated by immunohistochemistry (IHC) can be utilized as a surrogate marker for some *ARID1A* mutations, and it is correlated to MSI-H type and EBV positivity [71,72]. However, several studies reported higher percentages of *ARID1A* IHC losses compared to molecularly confirmed *ARID1A*-deficient cases [73,74,75]. These discrepancies could be accounted for by epigenetic silencing through promoter methylation or post-transcriptional modification which can also cause the loss of *ARID1A* expression [73,74,75]. Furthermore, a recent study suggested that EBV-encoded miRNA in EBV-positive GCs can regulate *ARID1A* expression [29]. As such, further studies are needed to clarify the range of mechanisms responsible for *ARID1A* silencing in GC.

**Figure 2:**
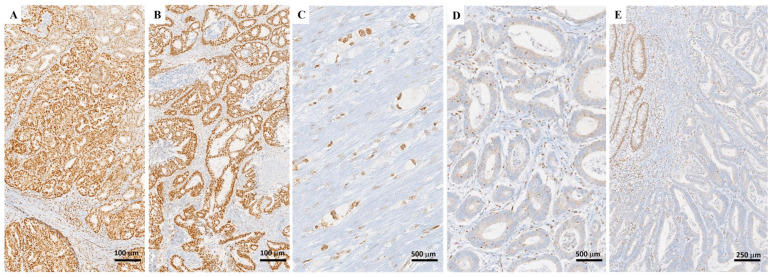
Immunohistochemical staining patterns of ARID1A (images taken from author’s pathological archives): (**A**,**B**) Diffuse nuclear staining for ARID1A in a case of intestinal-type tubular adenocarcinoma of the stomach is depicted. (**C**) Diffuse nuclear staining in a diffuse-type gastric carcinoma (poorly cohesive carcinoma). These stainings are considered positive. (**D**,**E**) Another example of tubular adenocarcinoma of the stomach showing negative staining for ARID1A is depicted. Positive ARID1A staining, observed in the stromal cells as well as non-neoplastic glands, served as a positive internal control.

According to existing literature, ARID1A expression in GC can be categorized either as positive (diffuse nuclear staining) or negative (complete nuclear loss of *ARID1A* expression, with positive stromal cells as internal controls) (Figure 2) [72]. However, recent studies emphasized that two additional staining patterns may be observed in a subset of tumors: heterogeneous (also called ‘checkerboard’ staining pattern) and ‘clonal loss’ pattern (presence of a neoplastic cell subpopulation within the tumor showing abrupt absence of nuclear staining) [73,74,75] (Table 4). Several studies have demonstrated that heterogeneous and clonal loss staining patterns are associated with mutations in *ARID1A* and therefore should be scored as ‘loss of expression’ [73,74,75]. In this regard, partial loss of ARID1A IHC (heterogeneous or clonal loss) has also been correlated with an increased expression of PD-L1 in GC cells and higher levels of PD-1+ TILs [73,74,75]. Furthermore, it is widely accepted that heterogeneous *ARID1A* loss is significantly correlated with the PIK3CA mutation [73,74,75]. Nevertheless, a recent study proposed that any *ARID1A* loss—irrespective of the percentage area of the tumor affected (heterogeneous/clonal/diffuse)—may be associated with specific clinicopathological or molecular features [76].

To date, the main limitations of ARID1A IHC relate to the variety of immunohistochemical assays (including antibodies) staining platforms, cut-offs, and scoring systems in use which likely explain the divergence in the reported percentages of *ARID1A*-deficient cases. It is widely known that intratumoral heterogeneity frequently occurs in GC, and this phenomenon may also account for the different expression patterns of ARID1A observed across published studies [72,73,74,75]. Therefore, *ARID1A* IHC assessment should ideally be performed in surgical resection specimens since smaller tissue specimens and tissue microarrays may lead to sampling errors [72,73,74,75].

Interestingly, spatial heterogeneity of ARID1A expression has also been reported to occur in GCs [75]. A recent study reported markedly different *ARID1A* staining patterns between primary tumors and their matched lymph node metastases [75]. In this study, patients with heterogeneous ARID1A expression in the primary tumor showed different lymph node metastases staining patterns as diverse as complete loss of ARID1A (53.2%), retained expression (21.3%), and heterogeneous expression (25.5%) [75].

## 8. *ARID1A* Loss in Precursor Lesions

Abnormalities of ARID1A have also been documented in precancerous lesions. In the study by Abe et al., the authors evaluated ARID1A loss in EBV-associated gastric carcinoma, by performing in situ hybridization of EBV-encoded RNA and immunohistochemistry of ARID1A in non-neoplastic gastric mucosa and intramucosal cancer tissue [77]. In this study, authors have shown that the absence of ARID1A is associated with specific morphological characteristics (tubular structure) in the mucosal layer. It also facilitates EBV infection in gastric epithelial cells, suggesting its potential role in initiating viral-driven carcinogenesis [77]. In a subsequent study by the same authors, ARID1A loss was investigated by immunohistochemistry in early gastric cancer and non-neoplastic gastric mucosa [78]. ARID1A loss was detected in 10% of non-neoplastic mucosa including pseudo-pyloric and intestinal metaplastic glands devoid of dysplastic changes [78]. Moreover, in early gastric cancer cases, ARID1A loss was frequently detected in EBV-associated gastric cancer cases [78]. Therefore, the authors concluded that epithelial cells lacking ARID1A can undergo malignant transformation through a distinct pathway compared to p53-deficient intestinal metaplasia. This progression typically involves one or more steps leading to the development of carcinoma, such as EBV infection [77,78].

## 9. Role of *ARID1A* in Development and Progression of Tumors Other Than Gastric Cancer

ARID1A, a member of the SWI/SNF chromatin remodeling complex, has gained increasing attention in cancer research due to its roles in tumor initiation and suppression in several tumor types other than gastric cancer [16].

- Hepatocellular Carcinoma (HCC): ARID1A is one of the most frequently mutated genes in hepatocellular carcinoma, with mutations occurring in 10% to 17% of cases. ARID1A mutations affect several pathways critical for tumor growth [16,79,80]. Low ARID1A expression correlates with shorter patient survival, suggesting its involvement in HCC development and metastasis [79,80,81].
- Endometrial Cancer: The rate of ARID1A mutation in low-grade endometrioid adenocarcinomas is 47%, while in high-grade endometrioid adenocarcinomas, serous adenocarcinomas, and carcinosarcomas, it is 60%, 11%, and 24%, respectively [16,82,83,84]. Moreover, in 14–22% of uterine endometrial clear cell carcinoma, ARID1A expression is also found to be downregulated [16,83,84]. Notably, ARID1A mutations have been reported to occur also in preneoplastic lesions, indicating its role in early cancer development. [16,83,84].
- Ovarian Cancer: The mutation rate of ARID1A in ovarian clear cell carcinoma and ovarian endometrioid carcinomas (OEC) is 46–57% and 30%, respectively [16,85,86]. Additionally, ARID1A is more frequently lost in mismatch repair deficient ovarian clear cell carcinoma [16,85,86,87].

ARID1A is also involved in the development of choriocarcinoma, where its overexpression of ARID1A suppresses migration and invasion of choriocarcinoma cells, while its inhibition promotes migration and invasion, suggesting a tumor-suppressor role of ARID1A in choriocarcinoma progression [16,85,88,89].

- Colorectal Cancer: ARID1A mutations are detected in 10% of colorectal cancers and are strictly related to mismatch repair deficiency [16,90,91]. In detail, ARID1A downregulation has been reported to influence the proliferation of colorectal cancer cells and their resistance to chemotherapy [16,90,91]. Moreover, ARID1A loss has been shown to promote epithelial–mesenchymal transition (EMT) in colon cancer, contributing to metastasis [16,90,91].
- Pancreatic Cancer: Recent comprehensive sequencing analyses of pancreatic cancer have demonstrated ARID1A mutations in 6% of cases [16,92,93]. ARID1A may represent a tumor suppressor gene in pancreatic carcinogenesis, as its expression levels correlate with tumor differentiation and stage, although not with lymph node or distant metastasis, sex, or age [16,92,93]. In mouse models, ARID1A deficiency has been shown to accelerate tumor progression, leading to high-stage disease [16,92,93].
- Breast Cancer: ARID1A not only exerts antitumor effects such as inhibiting cancer cell migration and invasion in breast cancer but also enhances the sensitivity of breast cancer cells to chemotherapy [16,94,95,96,97]. Moreover, it has been shown to influence the activity of estrogen receptor α+ [16,94,95,96,97]. This receptor, when activated, induces an oncogenic signal which regulates tumor cell proliferation in breast cancer [77,92,93,94,95]. Therefore, wild-type ARID1A has been shown to correlate with improved clinical outcomes in ER+ breast cancer patients [77,92,93,94,95]. By contrast, ARID1A inactivating mutations are more frequently detected in treatment-resistant and metastatic tumors [16,94,95,96,97].

## 10. Clinical Utility of ARID1A in GC: Limitations, Challenge and Future Directions

The clinical and prognostic significance of ARID1A in gastric cancer is still a matter of debate. Much of the current clinical evidence is based on small case series, which may introduce bias and other influencing factors. Additionally, due to the limitations in both single therapy resistance and combined treatment adverse reactions, careful monitoring of dosage and usage of ARID1A-mutant GC-targeted therapy drugs is essential. Furthermore, further research is needed to fully understand the role that ARID1A mutation plays in tumor formation, development, predictive response to treatments, as well as biological mechanisms. In this perspective, large-scale prospective clinical studies are needed to provide more useful insights into the predictive and prognostic value associated with ARID1A mutations in GC patients. Future studies should also prioritize examining the association between ARID1A deficiency and PD-L1 expression, building on previous studies that have found this link in various groups of patients. This could pave the way for a combined treatment approach targeting both PD-L1 and ARID1A.

## 11. Conclusions

There is mounting scientific and clinical evidence supporting the importance of, and the molecular mechanism underlying, *ARID1A* mutations in GC. *ARID1A* assessment by IHC may represent a potential prognostic biomarker related to several clinicopathological features, including tumor differentiation, nodal metastases, and specific GC TGCA subtypes. Moreover, *ARID1A* loss may contribute to predicting patient response to novel treatment strategies such as immunotherapy, PARP inhibitors, mTOR inhibitors, EZH2 inhibitors, and histone deacetylase inhibitors. Additionally, ARID1A alterations could be associated with resistance to platinum chemotherapy and estrogen receptor modulators [98]. Collectively, these findings suggest the utility of testing this gene either by IHC or by molecular techniques in order to expand our knowledge of its role in GC and to improve the therapeutic strategies available for GC patients.

In conclusion, in this comprehensive review, we have focused on detailing the clinical significance, predictive value, underlying mechanisms, and potential treatment strategies for ARID1A mutations in gastric cancer. Our primary aim was to contribute theoretical support for future research on utilizing ARID1A as a biomarker to stratify individuals with gastric cancer and enable precision therapy. We expect that our analysis will lead to improved clinical outcomes for the subset of patients afflicted by GC with an ARID1A mutation.

## Figures and Tables

**Table 1:** Clinical and prognostic significance of ARID1A mutation in gastric cancer.

Study	ARID1A Expression Status	Sample Size	OS	PFS	Prognostic Significance
Zhou et al. [20]	Lower than normal tissue	Not specified	Not specified	Reduced	Not specified
Wang et al. [29]	Loss.	272 primary GCs	Associated with poor prognosis	Not specified	Independent risk factor for poor prognosis
Ibarrola–Villava et al. [30]	Loss	Not specified	Higher than those with positive expression	Not specified	Challenges the association with poor prognosis
Wiegand et al. [31]	Loss	173 GCs	No clear relationship observed	Not specified	Conflicting findings

**Table 2:** ARID1A implications in gastric cancer.

		References
Frequency of ARID1A mutations in GC	- 8–27% of cases, predominantly in EBV-positive subtype - 44.2% in MSI - 13.9% in MSS/EMT - 18.6% in MSS/TP53+ - 5.9% in MSS/TP53-	Qadir et al. [32] Blanchet et al. [33] Reske et al. [34] Lei et al. [35]
Prognostic role of ARID1A	loss of ARID1A expression is associated with both reduced progression-free survival (PFS) and overall survival (OS)	Wang et al. [36] Yang et al. [37] Inada et al. [38] Kim et al. [29] Fontana et al. [39]
Interaction of ARID1A with other gene pathways	- TP53 - PIK3/AKT pathway	Zhang et al. [40] Guan et al. [41] Bosse et al. [42] Loe et al. [43]
Immune-related biomarkers related to ARID1A loss	- MSI - PD-L1 - TILs - TMB	Setia et al. [44] Kim et al [45] Carrasco et al. [46]
Therapeutic strategies in ARID1A-deficient GC	- PD-1/PD-L1 inhibitors - PARP inhibitors - mTOR inhibitors - PI3K inhibitors - AKT inhibitors	Lu et al. [47] Yang et al. [48] Bitler et al. [49] Yamada et al. [50]

**Table 3:** Therapeutic approach in ARID1A-deficient tumors.

Biomarker	Therapeutic Approach	Clinical Evidence	References/ Clinical Trials
PD-L1 Expression	Correlates with response to PD-1/PD-L1 inhibitors	KEYNOTE-059: Pembrolizumab effective in GC with PD-L1 CPS ≥ 1 CHECKMATE-649: Nivolumab + chemotherapy improved OS in GC/EGJ with PD-L1 CPS ≥ 5	NCT02335411 NCT02872116
Tumor Mutation Burden (TMB)	Predicts effectiveness of immunotherapy across tumor types	Pembrolizumab FDA approved for metastatic/unresectable solid tumors with dMMR or MSI-H biomarkers	Li et al. [67] Lemery et al. [65]
Mismatch Repair Deficiency	Significantly responds to immunotherapy	KEYNOTE-016, 164, 012, 028, and 158 trials	NCT01876511 NCT02460198 NCT01848834 NCT02054806 NCT02628067
Tumor-Infiltrating Lymphocytes	Potential biomarker for PD-1/PD-L1 immunotherapy success	Recognized for predicting PD-1/PD-L1 immunotherapy success	Angelico et al. [65]
ARID1A Expression	Correlates with PD-L1 expression, TMB, dMMR/MSI-H, and TILs	Associated with upregulation of PD-L1 via PI3K/AKT/mTOR pathway—Bioinformatics suggest ARID1A-mutated GC may benefit from immunotherapy	Kim et al. [66] Li et al. [67]
EZH2 Overexpression	Influences tumor-infiltrating lymphocytes and immunosuppression	Targeting EZH2 may enhance existing immunotherapies in ARID1A-mutated cancers	Lu et al. [47] Bitler et al. [49]

**Table 4:** Immunohistochemical interpretation of ARID1A in gastric cancer.

Staining Pattern	Interpretation	References
Diffuse nuclear staining	Positive: no ARID1A mutations	Guan et al. [72] Ye et al. [73] Bosse et al. [74] Saito et al. [75]
Complete nuclear loss of ARID1A expression	Negative: associated with mutations in ARID1A	
Heterogeneous ARID1A staining	Negative: associated with mutations in ARID1A	
Neoplastic cell subpopulation showing abrupt absence of nuclear staining	Negative: associated with mutations in ARID1A	
